# Retraction: Renal Trauma in a Solitary Kidney Due to Unilateral Renal Agenesis: A Case Report

**DOI:** 10.7759/cureus.r236

**Published:** 2026-07-21

**Authors:** Nabil Ettoumi, Nawfal Ettoumi, Hamza Ajjemami

**Affiliations:** 1 Urology, University Mohammed VI Polytechnic (UM6P) Hospitals, Benguerir, MAR; 2 Urology, Military Hospital Moulay Ismail, Meknès, MAR; 3 Radiology, Université de Picardie Jules-Verne, Amiens, FRA

This article has been retracted, and its contents removed, by the Editor-in-Chief due to concerns regarding the authors' permission to use and publish the clinical data presented. The authors were notified of the decision and agree with the retraction.

